# Association of SARS-CoV-2 viral load at admission with in-hospital acute kidney injury: A retrospective cohort study

**DOI:** 10.1371/journal.pone.0247366

**Published:** 2021-02-24

**Authors:** Ishan Paranjpe, Kumardeep Chaudhary, Kipp W. Johnson, Suraj K. Jaladanki, Shan Zhao, Jessica K. De Freitas, Elisabet Pujdas, Fayzan Chaudhry, Erwin P. Bottinger, Matthew A. Levin, Zahi A. Fayad, Alexander W. Charney, Jane Houldsworth, Carlos Cordon-Cardo, Benjamin S. Glicksberg, Girish N. Nadkarni

**Affiliations:** 1 The Mount Sinai Clinical Intelligence Center (MSCIC), Icahn School of Medicine at Mount Sinai, New York, New York, United States of America; 2 The Hasso Plattner Institute for Digital Health at Mount Sinai, Icahn School of Medicine at Mount Sinai, New York, New York, United States of America; 3 Department of Genetics and Genomic Sciences, Icahn School of Medicine at Mount Sinai, New York, New York, United States of America; 4 Icahn School of Medicine at Mount Sinai, The Charles Bronfman Institute for Personalized Medicine, New York, New York, United States of America; 5 Department of Anesthesiology, Perioperative and Pain Medicine, Icahn School of Medicine at Mount Sinai, New York, New York, United States of America; 6 Department of Pathology, Molecular and Cell-Based Medicine, Icahn School of Medicine at Mount Sinai, New York, New York, United States of America; 7 Icahn School of Medicine at Mount Sinai, BioMedical Engineering and Imaging Institute, New York, New York, United States of America; 8 Department of Diagnostic, Molecular and Interventional Radiology, Icahn School of Medicine at Mount Sinai, New York, New York, United States of America; 9 The Pamela Sklar Division of Psychiatric Genomics, Icahn School of Medicine at Mount Sinai, New York, New York, United States of America; 10 Division of Nephrology, Department of Medicine, Icahn School of Medicine at Mount Sinai, New York, New York, United States of America; Indiana University School of Medicine, UNITED STATES

## Abstract

**Background:**

Severe acute respiratory syndrome coronavirus 2 (SARS-CoV-2) and the associated Coronavirus Disease 2019 (COVID-19) is a public health emergency. Acute kidney injury (AKI) is a common complication in hospitalized patients with COVID-19 although mechanisms underlying AKI are yet unclear. There may be a direct effect of SARS-CoV-2 virus on the kidney; however, there is currently no data linking SARS-CoV-2 viral load (VL) to AKI. We explored the association of SARS-CoV-2 VL at admission to AKI in a large diverse cohort of hospitalized patients with COVID-19.

**Methods and findings:**

We included patients hospitalized between March 13^th^ and May 19^th^, 2020 with SARS-CoV-2 in a large academic healthcare system in New York City (N = 1,049) with available VL at admission quantified by real-time RT-PCR. We extracted clinical and outcome data from our institutional electronic health records (EHRs). AKI was defined by KDIGO guidelines. We fit a Fine-Gray competing risks model (with death as a competing risk) using demographics, comorbidities, admission severity scores, and log_10_ transformed VL as covariates and generated adjusted hazard ratios (aHR) and 95% Confidence Intervals (CIs). VL was associated with an increased risk of AKI (aHR = 1.04, 95% CI: 1.01–1.08, p = 0.02) with a 4% increased hazard for each log_10_ VL change. Patients with a viral load in the top 50^th^ percentile had an increased adjusted hazard of 1.27 (95% CI: 1.02–1.58, p = 0.03) for AKI as compared to those in the bottom 50^th^ percentile.

**Conclusions:**

VL is weakly but significantly associated with in-hospital AKI after adjusting for confounders. This may indicate the role of VL in COVID-19 associated AKI. This data may inform future studies to discover the mechanistic basis of COVID-19 associated AKI.

## Introduction

Severe acute respiratory syndrome coronavirus 2 (SARS-CoV-2) has affected millions of patients worldwide [1]. SARS-CoV-2 was initially believed to be a respiratory virus, but emerging evidences now show that it may affect multiple organ systems independently [2–4].

Acute kidney injury (AKI) is now emerging as a common complication in hospitalized patients with COVID-19. Up to 40% of hospitalized patients with COVID-19 develop AKI, and it is linked to substantially higher morbidity and mortality [5, 6]. In addition, recent studies have shown that COVID-19 is associated with a higher incidence of AKI compared to other infections, and patients with COVID-19 associated AKI are more likely to require acute renal replacement therapy, have higher rates of mortality, and less kidney function recovery compared to patients without COVID-19 [5, 7]. This has led to the hypothesis that COVID-19 associated AKI may be a distinct pathophysiological entity from other forms of AKI during acute, critical illness [8].

Several mechanisms, including direct viral kidney invasion and cytokine storm have been linked to increased SARS-CoV-2 viral load (VL) phenomenon; however, there is currently no consensus [9]. We have shown previously that SARS-CoV-2 VL is associated independently with risk of mortality [10]. We explored whether VL at admission is associated with increased risk of in-hospital AKI, after accounting for a competing risk of mortality, in a diverse group of hospitalized patients with COVID-19.

## Methods

### Cohort selection

We included patients hospitalized between March 13th 2020 and May 19th 2020 with SARS-CoV-2 confirmed by reverse transcriptase polymerase chain reaction (RT-PCR) of nasopharyngeal swab samples measured by the Roche cobas® 6800. Viral load of patients who tested positive was quantified using an assay developed by the Mount Sinai Health System clinical laboratory, All patients were admitted from five hospitals in the Mount Sinai Health System in New York City.

### Clinical data

All clinical data were extracted from the Mount Sinai institutional electronic health records (EHRs) with outcomes determined on or before the data freeze date of June 5^th^ 2020. We extracted this data from the EPIC CLARITY database using custom SQL code. Our EHR database consists of several tables that replicate the backend of our clinical EHR server. Briefly these tables include diagnoses, medication administration, problem list, patient demographics, and procedure codes. All data exists in structured fields with the exception of clinical notes. Individual terms from clinical notes were mapped to SNOMED concepts using a natural language processing engine. History of clinical comorbidities were determined using ICD-9/10 diagnostic codes (S1 Table). As per the KDIGO criteria, AKI was defined as a 50% increase in serum creatinine over baseline or a creatinine change of 0.3 mg/dL over a 48-hour period [11]. We defined AKI stage as per KDIGO guidelines. Baseline creatinine was considered as the most recent creatinine value within 7–365 days prior to admission. Following KDIGO guidelines, for patients who did not have a baseline value, we imputed a baseline creatinine based on a MDRD eGFR of 75 ml/min per 1.73m^2^ as done in previous work [12]. If patients had multiple VL measurements, the first VL within three days of admission was taken. Patients with VL measurements after three days of admission were excluded (N = 47) for a final 1,049 patients included. Age, sex, and laboratory measurements were extracted from our institutional EHR. Self-reported race and ethnicity were extracted from the intake forms in the EHR. Drug administration history during the hospital admission were extracted from the medication admission record available in the EHR. This record includes all medications administered during the admission. We categorized medications of interest by class (nonsteroidal anti-inflammatory medications, angiotensin converting enzyme inhibitors, and angiotensin receptor blockers). We computed the CURB-65 score at the time of admission. Blood urea nitrogen, systolic and diastolic blood pressures, and respiratory rate were directly extracted from the EHR. The confusion parameter of the CURB-65 score was abstracted using natural language processing of clinical notes from the time of admission as previously described [13]. Specifically, all identifiable SNOMED terms identifiable in each note was extracted using a standard natural language processing engine (NLP) and then queried for symptoms related to “mentally alert” (SNOMED: 248234008) or “oriented” (SNOMED: 247663003) as we have shown previously.

### Viral load quantification

Realtime RT-PCR was performed with the Quantifast Pathogen RT-PCR kit (Qiagen) in a Lightcycler 480 II (Roche) using the N2 primer from the CDC 2019-nCoV Real-Time RT-PCR Diagnostic Panel. All extraction and RT-PCR were performed in a CLIA certified laboratory in the Mount Sinai Health System. The limit of detection, defined as the concentration at which at least 95% of replicates test positive, was determined to be 5 genome copies/mL. Viral load was quantified using the delta Ct method by plotting a standard curve fitting a linear regression model with Ct values for known concentrations of viral RNA.

### Statistical analysis

Details of statistical analysis and results are provided in a STROBE checklist (S2 Table).

We log_10_ transformed VL to minimize skewness. Time-to-AKI was determined as the difference between time of AKI onset and VL measurement. Individuals who remained hospitalized at the time of data freeze were censored. We fit a Fine Gray competing risks model [14] with age, sex, race, history of chronic kidney disease, CURB-65 score at admission, in-hospital administration of nonsteroidal anti-inflammatory medications, angiotensin converting enzyme inhibitors, and angiotensin receptor blockers, and log_10_ transformed viral load as covariates. We performed a time-to-AKI analysis using a competing risks model where death was treated as a competing risk since individuals who died in their hospital course may have died of causes unrelated to AKI. Since these individuals might have developed AKI if they had not died, including death as a competing risk accounts for this group of patients. We associated viral load with maximal AKI stage in a multivariate regression model. All statistical analysis was performed using R version 3.6.3 [15]. For univariate comparisons, P-values were estimated using a Wilcoxon rank-sum test for continuous variables and Fisher’s exact test for categorical variables.

### Ethics statement

This research has been approved by the Institutional Review Board (IRB) of the Icahn School of Medicine at Mount Sinai. All data were patient level data and not anonymized. The IRB waived requirement for informed consent.

## Results

Individuals who developed AKI during their hospital course had a higher log_10_ mean VL at admission (5.07 vs 4.24, p<0.001), were significantly older (70 vs 63 years, p <0.001), more frequently Black (36% vs 27%, p = 0.003), and had a higher prevalence of history of diabetes (23% vs 16%, p = 0.002) and chronic kidney disease (14% vs 3%, p<0.001) as compared to patients who did not develop AKI (Table 1).

**Table 1:** Baseline characteristics of AKI cases and controls.

Characteristic	AKI Cases (N = 443)	AKI Controls (N = 606)	sP Value	Number of Patients with Measurement
**Age, Mean (SD)**	69.9 (14.3)	62.5 (15.9)	<0.001	1049
**Race, n (%)**				1049
White	112 (25%)	169 (28%)	0.003	
Black or African-American	161 (36%)	161 (27%)		
Asian	10 (2%)	26 (4%)		
Other	142 (32%)	225 (37%)		
Unknown	18 (4%)	25 (4%)		
**Ethnicity, n(%)**				1049
Hispanic/Latino	91 (21%)	151 (25%)	0.186	
Non-Hispanic/Latino	287 (65%)	361 (60%)		
Unknown	65 (15%)	94 (16%)		
**Male, n (%)**	272 (61%)	365 (60%)	0.760	1049
**Clinical Comorbidities, n (%)**				1049
Atrial Fibrillation	36 (8%)	34 (6%)	0.132	
Coronary Artery Disease	52 (12%)	63 (10%)	0.548	
Hypertension	152 (34%)	177 (29%)	0.080	
Asthma	21 (5%)	40 (7%)	0.230	
Diabetes	104 (23%)	96 (16%)	0.002	
Chronic Obstructive Pulmonary Disease	19 (4%)	21 (3%)	0.517	
Chronic Kidney Disease	62 (14%)	18 (3%)	<0.001	
Viral load at Admission—log10 (copies/mL), Mean (SD)	5.07 (2.96)	4.24 (3.01)	<0.001	1049
**Inflammatory Markers at Admission, mean (SD)**				
White Blood Cell Count, K/μL	8.78 (6.01)	6.99 (3.58)	<0.001	1039
Lymphocyte Count, K/μL	1.0 (0.54)	1.12 (0.764)	<0.001	1039
C-Reactive Protein, mg/L	151 (96.3)	120 (86.8)	<0.001	813
Procalcitonin, pg/mL	2.03 (11)	0.569 (4.05)	<0.001	655
D-Dimer, μg/mL	3 (3.28)	1.78 (2.2)	<0.001	675
Fibrinogen, mg/dL	639 (196)	648 (189)	0.500	345
Ferritin, ng/mL	1460 (1960)	1230 (1980)	0.004	805
**CURB65 at Admission, mean (SD)**	1.92 (1.06)	0.929 (0.927)	<0.001	1049

P-values were computed using the Fisher’s exact test and Wilcoxon rank-sum test for categorical and continuous variables, respectively.

**Fig 1:**
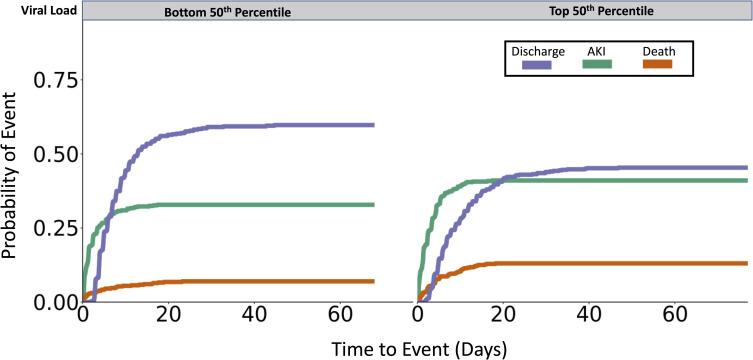
Cumulative incidence plot displaying probability of discharge, AKI, and death for individuals in the top and bottom 50^th^ percentile of viral load. Individuals who remained hospitalized at the time of data freeze were censored.

In a model adjusted for age, sex, history chronic kidney disease, admission CURB-65 score, in-hospital administration of nonsteroidal anti-inflammatory medications, angiotensin converting enzyme inhibitors, and angiotensin receptor blockers, VL increment was associated with an increased risk of AKI (HR = 1.04, 95% CI: 1.01–1.08, p = 0.02) with a 4% hazard increment for each log_10_ increment in VL. Individuals with a viral load in the top 50^th^ percentile had an increased adjusted hazard of 1.27 (95% CI: 1.02–1.58, p = 0.03) for AKI as compared to those in the bottom 50^th^ percentile (Fig 1). We then stratified cases by maximal AKI stage. We found the viral load associated with AKI stage in a dose dependent manner in a multivariate logistic regression model adjusted for the above covariates. We found the strongest association with AKI stage 3 (HR = 1.08, 95%CI: 1.02–1.15; p = 0.006), and suggestive associations with AKI stage 2 (HR = 1.08, 95% CI: 0.99–1.17, p = 0.07) and AKI stage 1 (HR = 1.06, 95% CI: 0.99–1.12, p = 0.08).

We then performed a sensitivity analysis by adjusting for inflammatory markers at the time of admission in addition to the above covariates. Individuals who developed AKI had a significantly greater C-reactive protein, procalcitonin, D-dimer, and ferritin and significantly lower lymphocyte count than controls (Table 1). After adding C-reactive protein, lymphocyte count, procalcitonin, D-dimer and ferritin to the fully adjusted model, we still found a significant association of VL with increased risk of AKI (HR = 1.06, 95% CI: 1.01–1.12, p = 0.02).

## Discussion

In a large, diverse group of patients with COVID-19, we report a significant adjusted association between admission VL and risk of in-hospital AKI. The association between VL and AKI was weak but significant and persisted after adjustment for both chronic comorbidities and admission severity of illness.

Although AKI has emerged as a common complication during the hospital course of COVID-19 [2, 6, 8], the mechanisms remain elusive. Recent autopsy data suggest that direct viral invasion may not be the likely primary mechanism of AKI [16]. However, there are contradictory studies which show that SARS-CoV-2 can be detected in multiple organs, with selective tropism for the kidneys, even in patients without a history of chronic kidney disease or those not critically ill [17]. Finally, a recent post-mortem study in 63 patients showed that SARS-CoV-2 renal tropism is associated with disease severity (i.e. premature death) and development of AKI [18].

We add to this literature by showing that admission VL independently associates with an increased risk of AKI in a competing risks framework. Importantly, we adjusted our analysis for the history of CKD since individuals with CKD may both have increased VL and be more predisposed to AKI. Since it is unknown whether individuals who died of other causes may have developed AKI if they had not died, it is necessary to account for death as an alternate outcome that precludes an individual from ever developing AKI. To account for this, we utilized a competing risks model with death as a competing risk. In this manner, our analysis partially accounts for the potential confounding of non-AKI related death.

Higher VL at admission may represent more severe disease and a strong proinflammatory state. However, we find that even after adjusting for inflammatory markers at the time of admission, VL is still significantly associated with risk of AKI. Thus, although an inflammatory milieu may contribute to AKI, VL has an independent role in driving AKI through an alternative mechanism.

Our work sheds light into the role of VL at admission and the risk of AKI. Recent work suggests that individuals with high VL at admission have an increased risk of death [10]. Our results similarly indicate that high VL is associated with AKI as a complication during the hospital course. We propose several potential explanations. Recent work has shown that high VL is associated with severe disease complications including septic shock and non-respiratory organ failure [19]. Thus, AKI may be secondary to renal hypoperfusion in the context of COVID-19 induced sepsis. However, the association of VL and AKI persists despite adjusting for baseline COVID-19 severity. Second, high VL may reflect impaired host immune response and thus is a predictor of risk of acute complications. In addition, whether host immune response is accurately estimated by viral load remains a testable hypothesis. Third, individuals with high VL at the time of admission may have higher *ACE2* receptor expression in the kidney, allowing for greater viral renal tropism. Recent work has shown that VL correlates with lung disease severity [20], likely mediated by direct viral entry through the ACE2 receptor expressed in the lung. Similarly, through an interplay between host immune response and local renal *ACE2* receptor expression, viral load may be an indicator for renal tropism. However, correlation with autopsy specimens is required to investigate this hypothesis. Finally, it is possible that high VL leads to a cytokine release syndrome, contributing to organ injury [21]. These hypotheses need to be examined using biomarker studies in prospective cohorts as well as mechanistic models of disease.

Our work should be interpreted in the light of certain limitations. First, VL may be influenced by certain medications such as corticosteroids administered before hospitalization. Second, although we adjusted for potential confounding, there may be unmeasured confounders that may have influenced our results. Third, since individuals presented to the hospital at different timepoints in their disease course, the VL measurements included in our study may or may not represent the peak VL for each individual and do not represent the VL at time of initial infection. Each patient’s true peak VL may correlate better with degree of kidney injury. Additionally, after resolution of AKI, whether VL decreases as viral particles are cleared remains an unanswered question. Large prospective studies with serial measurement of VL to determine peak VL are needed. Our cohort was selected to include all patients who were hospitalized with a positive COVID-19 test without strict inclusion and exclusion criteria to limit selection bias. However, since our cohort only included patients from New York City where resources were limited during the peak of the COVID pandemic, these results may not be generalizable and will need to be validated in other cohorts. Additionally, controls in our dataset were defined as all individuals who did not develop AKI and thus may have had other comorbidities or hospital complications.

In summary, we demonstrate an independent, significant association with admission VL and in-hospital AKI. This work may open new avenues to understanding the pathophysiological and mechanistic basis of COVID-19 associated AKI.

## Supporting information

S1 Table Diagnosis codes used to identify comorbidities. Individual diagnosis codes were extracted from each patient’s electronic health record. Patients were identified as having a specific comorbidity if they had at least one of the corresponding diagnosis codes in their record.
(CSV)

S2 Table STROBE checklist. (DOCX)
